# In vitro human cell-based TTR-TRβ CALUX assay indicates thyroid hormone transport disruption of short-chain, medium-chain, and long-chain chlorinated paraffins

**DOI:** 10.1007/s00204-021-02994-5

**Published:** 2021-02-08

**Authors:** Jannik Sprengel, Peter A. Behnisch, Harrie Besselink, Abraham Brouwer, Walter Vetter

**Affiliations:** 1 Institute of Food Chemistry (170B), University of Hohenheim, Garbenstr. 28, 70599 Stuttgart, Germany; 2 BioDetection Systems B.V. (BDS), Science Park 406, 1098 XH Amsterdam, Netherlands

**Keywords:** Polychlorinated *n*-alkane, Chloroparaffin, Thyroid disruption, Countercurrent chromatography, Thyroxine, Bioassay, TTR-TRβ CALUX

## Abstract

Over the last decades, short-chain chlorinated paraffins (SCCPs), medium-chain chlorinated paraffins (MCCPs), and long-chain chlorinated paraffins (LCCPs) have become the most heavily produced monomeric organohalogen compound class of environmental concern. However, knowledge about their toxicology is still scarce, although SCCPs were shown to have effects on the thyroid hormone system. The lack of data in the case of MCCPs and LCCPs and the structural similarity with perfluoroalkyl substances (PFAS) prompted us to test CPs in the novel TTR-TR CALUX assay for their thyroid hormone transport disrupting potential. Four self-synthesized and additionally purified single chain length CP mixtures (C_10_-CPs, C_11_-CPs, C_14_-CPs and C_16_-CPs) and two each of industrial MCCP and LCCP products were tested in parallel with PFOA. All CP mixtures influenced the TTR binding of T4, giving activities of 1,300 to 17,000 µg/g PFOA equivalents and lowest observable effect concentrations (LOELs) of 0.95 to 0.029 mM/L incubate. Highest activities and lowest LOELs were observed for C_16_-CPs (48.3% Cl content, activity 17,000, LOEL 0.047 mM/L) and a LCCP mixture (71.7% Cl content; activity 10,000; LOEL 0.029 mM/L). A trend of higher activities and lower LOELs towards longer chains and higher chlorination degrees was implied, but could not be statistically confirmed. Irrespectively, the less well examined and current-use LCCPs showed the highest response in the TTR-TRβ CALUX assay.

## Introduction

Chlorinated paraffins (CPs) are a group of current-use anthropogenic organohalogenated chemicals ubiquitously detected in the environment (Glüge et al. 2016, 2018; van Mourik et al. 2016). CPs are industrially produced by the chlorination of mixtures of alkane stocks to a desired degree of chlorination of typically 42, 52, or 70 wt.% (Sprengel and Vetter 2019; Glüge et al. 2018). Irrespective of chain length range and chlorination degree, each of the resulting technical CP mixtures consists of thousands of homologues whose individual structures are widely unknown. According to the ranges of alkane chains, they are subdivided into short-chain CPs (SCCPs, C_10–13_), medium-chain CPs (MCCPs, C_14–17_) and long-chain CPs (LCCPs, > C_17_). SCCPs were shown to be persistent, bioaccumulative and toxic, and thus considered as environmentally harmful compounds. Consequently, SCCPs were added to Annex A of the Stockholm Convention in 2017 (Conference of the Parties to the Stockholm Convention 2017). Given the structural similarity of SCCPs with MCCPs and LCCPs—which are only differing by chain lengths—similar detrimental environmental properties could be shown by the presence of (mainly) MCCPs in a wide range of samples (He et al. 2019; Wu et al. 2019; Hilger et al. 2013; Wang et al. 2019; Li et al. 2017). Arguably, this similarity in structure and environmental properties could also be valid in the case of toxicity. However, toxicological data on MCCPs and especially LCCPs are scarcely found in the scientific literature. For instance, SCCPs were shown to reduce levels of the thyroid hormones triiodothyroxine (T3) and thyroxine (T4) (Gong et al. 2018), causing developmental defects in zebrafish (Liu et al. 2016) and cancer in mice and rats (Wyatt et al. 1993). Compared to that only one technical MCCP product (40% Cl) was shown to interfere with thyroid hormones similar to SCCPs (Wyatt et al. 1993), and no data existed on the thyroid disruptive potential of LCCPs. Recently, poly- and perfluoroalkyl substances (PFAS) like perfluorooctanoic acid (PFOA) were shown to competitively bind to the thyroid transport protein transthyretin (TTR), which can lead to reduced thyroid hormone levels (Weiss et al. 2009). Moreover, a fast and reproducible cell line-based CALUX bioassay was introduced which enabled monitoring of thyroid hormone transport disruption by combining a TTR-binding assay with a TRβ CALUX detection assay (TTR-TRβ CALUX) (Collet et al. 2019). The strong dose-dependent TTR-binding potency of PFOA could be confirmed in this bioassay (Collet et al. 2019). Given the structural similarity with PFOA (both consisting of long, unbranched carbon chains with a high level of halogenation), we reasoned that CPs may also give response in the TTR-TRβ CALUX bioassay and that this test could be used to monitor thyroidal responses induced by CPs.

Hence, the goal of this study was to assess the thyroid disruptive potential of CP products by means of the TTR-TRβ-CALUX bioassay initially developed for polyhalogenated compounds. For this purpose, four technical CP products and four self-synthesized single chain length CP mixtures were used as substrates (Sprengel et al. 2019). Due to the possible presence of impurities in these products (Heeb et al. 2020), all single chain length CP mixtures were initially purified from potential impurities by means of countercurrent chromatography (CCC). CCC is a preparative all-liquid based instrumental method (Ito 2005) that is widely used for the isolation of natural products (Friesen et al. 2015). However, its merits were also shown in the fractionation of mixtures of polyhalogenated compounds (Gallistl and Vetter 2016; Kapp and Vetter 2009; Vetter et al. 2012). Dose ranges of CP products and mixtures were incubated with TTR and T4 for binding competition. Thereafter the remaining T4 bound to TTR was analyzed using the TRβ reporter assay. All CP products showed thyroid hormone disruptive potential in the TTR-TRβ CALUX bioassay.

## Materials and methods

### Industrial CP products and single chain length CP mixtures

The four industrial CP products were produced in China. Two consisted almost exclusively of MCCPs and the remaining two of LCCPs. Following their recent characterization by gas chromatography with electron capture negative ion mass spectrometry (GC/ECNI-MS) and nuclear magnetic resonance spectroscopy (NMR) (Sprengel and Vetter 2020), these were labeled A-52 (MCCP-1), B-52 (MCCP-2), A-42 (LCCP-1) and A-70 (LCCP-2) with the initial letter indicating two producers (A and B) and the number representing the approximate degree of chlorination in weight% (namely 42%, 52% or 70%) (Sprengel and Vetter 2020). Four self-synthesized single chain length CP mixtures were tested as well. Namely, a C_10_-CP mixture with 49.3% Cl content and a C_11_-CP mixture with 49.8% Cl content were selected as representatives for SCCPs and a C_14_-CP mixture with 50.1% Cl content and a C_16_-CP with 48.3% content Cl were chosen to represent the MCCP range (Sprengel et al. 2019). Recently, it was found that the synthesis route of Sprengel et al. (2019) might contain sulfur-containing impurities (Heeb et al. 2020). To exclude that these could affect the measurements, the four single chain CP mixtures were further purified by CCC before they were applied to the TTR-TRβ CALUX bioassay (section Clean-up of self-synthesized CPs via countercurrent chromatography (CCC)). Of all CP samples ~ 12 mg were used for the assay.

### Clean-up of self-synthesized CPs via countercurrent chromatography (CCC)

CCC fractionation of single chain CP mixtures was performed with an AECS QuikPrep MK8 instrument (London, U.K.) using the standard setup and the BTF solvent system (*n*-hexane/acetonitrile/α,α,α-trifluorotoluene (20:13:7, v:v:v)) of Englert et al*.* (Englert et al. 2015). Coil 1 (123 mL) was used for the separation. After filling the coil with stationary phase, rotation was set to the maximum value of 870 rpm and mobile phase was pumped into the system at 10 mL/min until its breakthrough and stabilization of the system (retention of the stationary phase: S_f_ = 75%). Then, the flow rate was decreased to 3.5 mL/min and ~ 500 mg of the respective CP product, dissolved in 10 mL upper phase, was injected into the CCC system. The first 130 mL (C_10_-CPs), 150 mL (C_11_-CPs), 180 mL (C_14_-CPs) or 190 mL (C_16_-CPs) were collected as one fraction containing the more polar sulfur-containing impurities. After that, 10 fractions of 7 mL each were collected, respectively. Then, the coil was purged with methanol at 10 mL/min for displacement of the stationary phase which was also collected. Individual CCC fractions were evaporated to dryness, weighed and re-dissolved with *iso*-octane (for pesticide residue analysis grade) to give concentrations of ~ 200 ng/µL. These solutions were analyzed by GC/ECNI-MS analysis to check for purity of the fraction. Sulfur-containing impurities were monitored by GC/ECNI-MS operated in full scan mode by extraction of *m/z* 99 and 101 ([SO_2_Cl]^−^) from the total ion current which were additionally requested to be present in the ratio 2.68:1. CCC fractions giving response were removed and those of CPs without impurities were pooled (Table 1).

**Table 1:** Impurity-free CPs gained from CCC clean-up of self-synthesized single chain length CP standards

CP standard	Chlorination degree of raw product (%)	Amount of raw product (mg)	Start of pure CP fraction (mL)	Amount of pure CP fraction (mg)
C_10_-CP (49.3.% Cl)	50.3	487	151	173
C_11_-CP (49.8% Cl)	51.2	494	164	333
C_14_-CP (50.1% Cl)	51.8	640	187	177
C_16_-CP (48.3% Cl)	50.9	269	218	234

### TTR-TRβ CALUX^©^ assay of CP mixtures and perfluorooctanoic acid (PFOA)

The TTR-TRβ CALUX bioassay was carried out by BioDetection Systems B.V. (BDS, Amsterdam, the Netherlands) under conditions described in detail previously (Collet et al. 2019). After dissolving 10–13 mg of CPs (Table 2) in 100 µL of DMSO, serial dilutions of all samples were prepared in DMSO in log-scale increments of 0.5. Five (5) µL of diluted CP samples were mixed with 100 µL TTR and 50 µL T4 (final concentrations 0.058 µM and 0.052 µM, respectively), both dissolved in Tris buffer (pH 8.0), and incubated in a final volume of 155 µL overnight at 4 °C. No precipitation of CPs was observed. TTR-bound T4 was separated from unbound (free) T4 by loading the total incubate on a pre-cooled Bio-Gel P-6DG column followed by 1 min centrifugation (210 g). One hundred and forty (140) µL of the collected eluate containing the TTR-bound T4 were mixed with 500 µL serum-free assay medium and added to seeded and pre-incubated TRβ CALUX cells (200 µL/well; in triplicate; Table 3). After 24 h of exposure of the TRβ CALUX cells in a conditioned environment (37 °C, 5% CO_2_, 100% humidity), the medium was removed and the cells were lysed with 30 µL of a triton-lysis buffer. The induction of the luciferase production was quantified after addition of 100 µL illuminate solution containing 15 µg of the substrate D-luciferin and subsequent measurement of luminescence on a luminometer (Mithras LB949, Berthold Technologies, Bad Wildbach, Germany). As standard reference substance, a serial dilution series of PFOA (Sigma-Aldrich, Zwijndrecht, The Netherlands) in DMSO in log-scale increments of 0.5 was prepared (4.6 × 10^–2^–1.3 × 10^–5^ mol/L) and 5 µL of each dilution was incubated with 50 µL T4 and 100 µL TTR.

**Table 2:** TTR-TRβ CALUX^©^ PC_80_, IC_50_ values as well as thyroid hormone displacement activity (TTR-T4_disp_) and lowest observed effect concentration (LOEC) of four self-synthesized single chain and four technical CP mixtures

Sample	Mean carbon formula [mean molar mass (g/mol)]	Amount used in stock solution (mg)	PC_80_ (mmol/L incubate)	IC_50_ (mmol/L incubate)	TTR-T4_disp_ activity (µg PFOA equivalents/g)	LOEC (mmol/L incubate)
PFOA	(414)	–	8.15E−04	0.00204	–	
C_10_-CP (49.3.% Cl)	CH_1.82_Cl_0.38_ (273)	13.2	0.97	(–)	1300	0.95
C_11_-CP (49.8% Cl)	CH_1.77_Cl_0.41_ (311)	10.7	0.30	0.65	3600	0.30
C_14_-CP (50.1% Cl)	CH_1.75_Cl_0.39_ (384)	12.3	0.053	0.21	9200	0.094
C_16_-CP (48.3% Cl)	CH_1.74_Cl_0.36_ (427)	11.7	0.047	0.18	17,000	0.047
MCCP-1 (53.1% Cl)	CH_1.68_Cl_0.44_ (440^a^)	12.2	0.10	(–)^b^	4200	0.18
MCCP-2 (52.3% Cl)	CH_1.68_Cl_0.44_ (441^a^)	12.9	0.36	(–)^b^	2100	0.36
LCCP-1 (41.1% Cl)	CH_1.86_Cl_0.26_ (578^a^)	12.3	0.25	(–)^b^	2300	0.24
LCCP-2 (71.7% Cl)	CH_1.20_Cl_0.93_ (1150^a^)	12.2	0.029	(–)^b^	10,000	0.029

^a^Exact chain length composition of the technical standards was unknown; therefore, the mean value of the contained chain lengths was chosen as an approximation: C_15_ for MCCPs (C_14–17_) and C_25_ for LCCPs (C_20–30_)

^b^IC_50_ could not be determined because of biphasic behavior

**Table 3:** CALUX^©^ cell culture and exposure medium information for the TRβ assay

Assay	TRβ
Cell type	U2-OS
Species	Human
DMSO	0.1%
Fold dilution	1000
CO_2_	5%
Exposure time	24 h
Seeding concentration	100,000 cells/mL (10,000 cells/well)
Medium used	DMEM/F-12
Additions to medium	-Stripped fetal calf serum (cell culture only) -Non-essential amino acids

The thyroid hormone disruptive potential (activity) of tested substances was determined relative to the standard reference substance PFOA from the concentrations giving a decrease in TRβ activation of 20% (PC_80_). Results were interpolated in the calibration curve for quantitative determination of thyroid hormone transport disruptive potential using the statistical software package GraphPad Prism V5.03 (STATCON, Witzenhausen, Germany), and further statistical test were performed in IBM SPSS Statistics 26 (Armonk, NY, USA). Where appropriate, the 50% maximal inhibitory concentration (IC_50_ value) was calculated.

## Results

CCC separation of the self-synthesized single chain length CP mixtures effectively removed all detectable amounts of sulfur-containing impurities from the raw products. The resulting, pure CP fractions showed smaller chlorination degrees than the raw mixtures (Table 1). Note, however, that the separated fractions were still dominated by CPs, and that the share of CPs in the raw product was much higher as may be concluded from the difference in sample weights before and after CCC as shown in Table 1. However, it was considered essential that any by-products were removed before the tests.

**Fig. 1:**
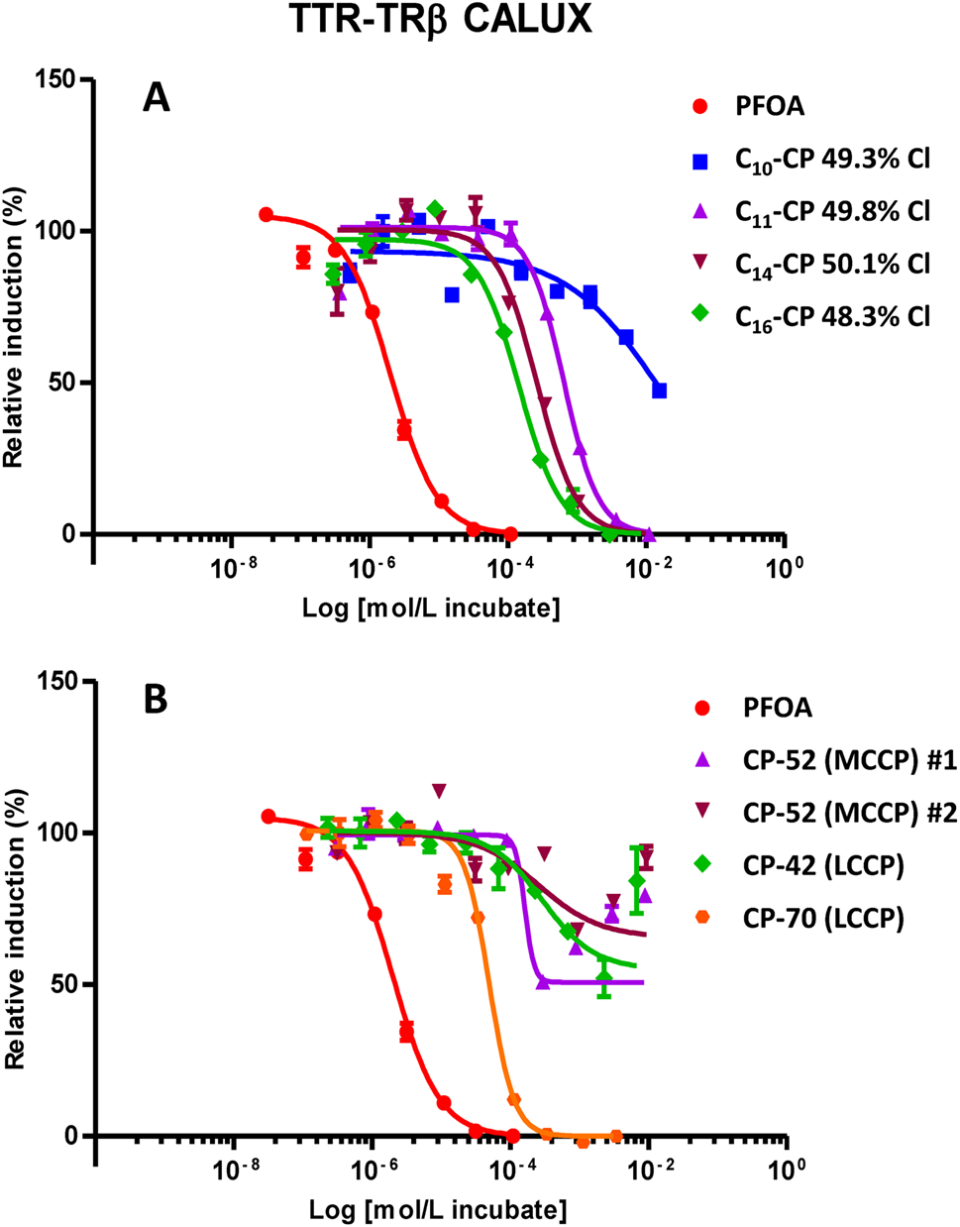
Dose–response TTR-T4 displacement (TTR-T4_disp_) curves of four self-synthesized single chain length CP standards (**a**) and four technical CP products (**b**) in the TTR-TRβ CALUX assay

All eight CP samples showed activity in the TTR-TRβ-CALUX^©^ (expressed as µg PFOA equivalent per g substance), indicating thyroid hormone transport disrupting potential (Table 2). The four single chain length CP mixtures showed similar chlorine contents (48.3–50.1% Cl content, section Industrial CP products and single chain length CP mixtures) and therefore similar mean carbon formulas (Sprengel and Vetter 2020). However, the TTR-T4 displacement (TTR-T4_disp_) in the TTR-TRβ-CALUX^©^ test increased in the order C_10_- < C_11_- < C_14_- < C_16_-CPs by more than one order of magnitude (from 1300 up to 17,000, Table 3). Hence, the response increased with the length of the alkyl chain, which indicated a higher activity of MCCPs in the test compared to SCCPs. All single chain CP mixtures showed a dose–response effect, with only C_10_-CPs not completely displacing T4 from TTR at the highest measured concentration (Fig. 1).

Also, the four industrial CP mixtures showed activity in the test. Two industrial MCCP mixtures from two producers showed similar %Cl content and also the activity in the bioassay was in a similar range (Table 2). A high response was also observed for the two technical LCCP products. In this case, the activity of the highly chlorinated mixture (71.7% Cl vs. 41.1% Cl) was more than fourfold higher (Table 3). Since the chain length composition of LCCPs could not be studied by GC/MS (chain lengths > C_18_ are difficult to analyze by GC (Schinkel et al. 2018)), it remained unclear if the dissimilarity in response was solely due to different degrees of chlorination or also affected by chain length composition. Irrespective of this uncertainty, the highly chlorinated industrial product LCCP-2 showed the second highest activity of the eight tested CP mixtures. Also, sample LCCP-2 showed the lowest observed effect level (LOEL) (Table 2) of all samples. In addition, LCCP-2 was the only industrial CP mixture that showed a dose–response effect like the single chain length CP mixtures (Fig. 1).

## Discussion

Overall, the individual responses of the tested CPs varied by one order of magnitude and could not be traced back to specific properties of the samples. However, response of all tested CPs in the TTR-TRβ CALUX test indicated that all types of CPs (SCCPs, MCCPs and LCCPs) may possess thyroid disruptive potential. To our knowledge, this is the first report on possible toxic effects of LCCPs. Comparison of single chain CP mixtures of similar %Cl (at ~ 48–50% Cl) indicated increasing activity and lower LOELs with increasing chain length (**Table ****3**). The two industrial MCCP products were dominated by C_14_-congeners (share > 60%). Still, the activity was lower than the one of the single chain CP mixtures. This indicated that other factors, e.g. specific but currently unknown structural units, could also play a role. However, neither chlorination degree or chain length nor CALUX activity or LOEL was found to be significantly correlated (p >  > 0.05 for Pearson, Kendall-Tau and Spearmans Rho). Overall, limited information on the composition of CP mixtures prevented to determine the factors that were responsible for the different activities of the CP mixtures studied.

Irrespectively, reduced thyroid function as indicated in our tests can be harmful for the development of newborn babies (Spiliotis 2007). Recent studies described rising CP concentrations in mother’s milk (Zhou et al. 2020; Liu et al. 2020), from which they can be transferred efficiently to the newborn (Liu et al. 2020). The observation of thyroid disruptive activity of CPs in the TTR-TRβ-CALUX^©^ test may indicate a potential for thyroid hormone disruption when transferred from the mother to the infant. Hence, CP amounts in the diet of mothers should be as low as possible. Also, CPs were found to be present in commercial baby food (Krätschmer et al. 2021). Furthermore, additional thyroid toxicity tests featuring more different, but ideally, well-characterized CP mixtures are needed to clarify the factors that influence the different activity determined in the TTR-TRβ-CALUX^©^ test.
